# Latching dynamics in neural networks with synaptic depression

**DOI:** 10.1371/journal.pone.0183710

**Published:** 2017-08-28

**Authors:** Carlos Aguilar, Pascal Chossat, Martin Krupa, Frédéric Lavigne

**Affiliations:** 1 Bases, Corpus, Langage, UMR 7320 CNRS, Université de Nice - Sophia Antipolis, 06357 Nice, France; 2 Laboratoire J.A.Dieudonné UMR CNRS-UNS 7351, Université de Nice - Sophia Antipolis, 06108 Nice, France; 3 MathNeuro team, Inria Sophia Antipolis, 06902 Valbonne-Sophia Antipolis, France; 4 Department of Applied Mathematics, University College Cork, Cork, Ireland; McGill University Department of Physiology, CANADA

## Abstract

Prediction is the ability of the brain to quickly activate a target concept in response to a related stimulus (prime). Experiments point to the existence of an overlap between the populations of the neurons coding for different stimuli, and other experiments show that prime-target relations arise in the process of long term memory formation. The classical modelling paradigm is that long term memories correspond to stable steady states of a Hopfield network with Hebbian connectivity. Experiments show that short term synaptic depression plays an important role in the processing of memories. This leads naturally to a computational model of priming, called latching dynamics; a stable state (prime) can become unstable and the system may converge to another transiently stable steady state (target). Hopfield network models of latching dynamics have been studied by means of numerical simulation, however the conditions for the existence of this dynamics have not been elucidated. In this work we use a combination of analytic and numerical approaches to confirm that latching dynamics can exist in the context of a symmetric Hebbian learning rule, however lacks robustness and imposes a number of biologically unrealistic restrictions on the model. In particular our work shows that the symmetry of the Hebbian rule is not an obstruction to the existence of latching dynamics, however fine tuning of the parameters of the model is needed.

## Introduction

Prediction of changes in the environment is a fundamental adaptive property of the brain [1–5]. To this aim, the neural mechanisms subtending prediction must activate in memory potential future stimuli on the basis of preceding ones. In nonhuman primates processing sequences of stimuli, neural activity shows two main dynamics triggered by the presentation of the first stimulus (prime) that precede the second stimulus (target). First, some neurons strongly respond to the first stimulus and exhibit a retrospective activity at an elevated firing rate after its offset [1, 2, 6]. Retrospective activity is considered as a neural mechanism of short-term maintenance of the first stimulus in working memory [7–10]. Second, some neurons exhibit an elevated firing rate during the delay between the prime and target, i.e. before the onset of the target, and respond strongly to this target [1, 2, 11–18]. Prospective activity depends on previous learning of the pairs of prime and target stimuli [1, 2, 15, 16, 18–22] and is considered as a mechanism of prediction of the second stimulus [23–25]. Further, prospective activity of neurons coding for a stimulus is related to response times to process this stimulus when it is presented [15, 26]. In humans processing sentences, the EEG signal correlates with the level of predictability of target words from preceding prime words [27–31] (see [32] on fMRI and [33] on MEG signals). The early stages of processing of a word are facilitated when this word is predictable [27, 34, 35]) leading to a shorter processing time [36]. This so called priming of a target stimulus by a related preceding prime is reliably reported in both human [37–41] and nonhuman primates [15]; see [25] for a review. Further, experiments show that the magnitude of priming highly relies on the relation between the two stimuli stored in memory [29, 42–45] and on the overlap between features of the stimuli in memory [46, 47]. In both human and non-human primates, the relation between two stimuli stored in memory depends on the learned sequences of stimuli [42, 48, 49].

Many neurophysiological studies have described learning at the synaptic level as combinations of long-term potentiation (LTP) and long-term depression (LTD) of synapses [50–53]). On this basis, synaptic efficacy is an essential parameter to code the relation between stimuli in memory (e.g., [54, 55]). Further, single cell recordings and local field potentials report that neurons in the macaque cortex respond to several different stimuli [56–58]) and that a given stimulus is coded by the activity of a population of neurons [59–61]. As a result, the information about a specific stimulus is distributed across a pattern of activity of a neural population [62, 63]. Two different patterns of activity corresponding to two stimuli can therefore share some active neurons. Hence, such pattern overlap in the populations responsive to different stimuli can code a relation between these stimuli [64, 65].

Computational modelling studies of biologically inspired neural networks have been carried out in the context of the dynamics of neural activity in priming protocols used in human and nonhuman primates. Models show that retrospective activity of a stimulus is possible for high values of synaptic efficacy between neurons that are active to code for this stimulus [66, 67] and that prospective activity of a stimulus not yet presented is possible for high values of synaptic efficacy between neurons coding for the first stimulus and neurons coding for the second stimulus [68, 69]. On this basis, computational models have shown how a large spectrum of priming phenomena depends on the level of prospective activity of neurons coding for the second stimulus [25, 70, 71]. Taken as a whole, models have emphasised the essential role of the matrix of synaptic efficacies for the generation of specific levels of prospective activity generating specific levels of priming.

There have been a number of computational studies focussed on priming generated by the dynamics of populations of neurons with a distributed coding of the stimuli in attractor network models [72–76]. When presented with an external stimulus, these attractor networks converge to a stable steady state and do not activate a sequence of patterns. However, latching dynamics have been described as the internal activation of a sequence of patterns triggered by an initial stimulus [77] (see also [78–82]). In a model recently introduced [70, 71], several priming effects involved in prediction can be reproduced by latching dynamics that depend on the overlap between the patterns. This is made possible due to units that do not maintain constant firing rates, allowing the network to change state instead of converging to a fixed-point attractor. Interestingly, latching dynamics relies on the specific neural mechanisms of neural noise and fast synaptic depression. Neural noise is a fundamental property of the brain [83–85]). One of its functions is to increase the probability of state transitions in attractor networks [86, 87]. However, noise alone does not allow regular sequences of state transitions according to pattern overlap (see [88]). Fast synaptic depression reported in cortical synapses [89] rapidly decreases the efficacy of synapses that transmit the activity of the pre-synaptic neuron. A consequence is that the network cannot sustain a stable regime of activity of the neurons in a given pattern and spontaneously changes state. Connectionist models have shown the effects of fast synaptic depression on semantic memory [90] and on priming [70, 71]. When a stimulus is presented to the network, neurons activated by the stimulus activate each others in a pattern, but fast synaptic depression contributes to their deactivation because they activate each other less and less. In the meantime, these neurons begin to activate neurons of a different but overlapping pattern, that, because they are less activated, exhibit less synaptic depression at their synapses. Before fast synaptic depression takes its effect, the newly activated neurons can strongly activate their associates in the new pattern. The transition from the old to the new pattern is enabled by the synaptic noise. Hence the combination of neural noise and fast synaptic depression makes latching dynamics possible in attractor neural networks. However, the precise role of each of these mechanisms in changing the network state are still unclear. Further, the necessary and sufficient pattern overlap for latching dynamics and how it combines with synaptic depression and noise are still unknown. The aim of the present approach is to analyze the necessary and sufficient conditions of combination of neural noise, fast synaptic depression and overlap for the existence of latching dynamics, using the framework of heteroclinic chains [91].

Before introducing heteroclinic chains we would like to point to a number of works where a combination of synaptic depression and noise are used to study switching between different memory states, that is latching dynamics [92–95]. In our work we use a deterministic law for the evolution of synaptic depression, which results in monotonic decrease of the synaptic variable. This approximation requires a mean field limit. The authors of [92–95] use a formulation close to the mean field limit yet allowing for non-monotonic dependence of the coupling function on the firing rate patterns coding for the concepts. As a consequence the resulting latching dynamics can be chaotic. In some other works not only synaptic depression, but also facilitation is included in the model [96]. The novelty of our approach is the use of the concept of heteroclinic chains and singular perturbation theory, allowing for accurate prediction of the time and direction of the switches.

The term *heteroclinic chain* refers to a sequence of steady states joined by connecting trajectories. Heteroclinic chains or cycles have been studied in various contexts, including fluid dynamics, population biology, game theory and neuroscience (see [97–99] for a review), in particular in a model of sequential working memory [100]. Typically such chains involve states of saddle type, acting as sink for some trajectories and source for other ones. Latching dynamics, as investigated in the context of a Hopfield network [70, 71], strongly suggest a link to heteroclinic chains; similar dynamic behaviour has been found for networks of integrate and fire neurons with the same connectivity (learning) rule [101]. Following the set-up of [70, 71] we use Hopfield networks as attractor network models, however, following [91], we make a small change in the equation defining the network, in order to ensure that heteroclinic chains can exist in a robust manner. Another difficulty is that latching dynamics does not fit into the classical context of heteroclinic chains, as the learned patterns that lose stability due to synaptic depression cannot be seen as states of saddle type. Hence we need to consider generalized heteroclinic chains given as a sequence of connecting trajectories joining attractors (learned patterns) which become unstable due to a slowly varying variable (synaptic strength). The context of heteroclinic chains has the simplicity which allows for the derivation of numerous algebraic conditions that need to be satisfied in order for such chains (and hence latching dynamics) to exist. Therefore our work leads to a better qualitative and quantitative understanding of latching dynamics, including the role of overlap, synaptic depression, noise and feedback inhibition.

## Models

### The evolution of activity variables/firing rates

As in [70, 71] with somewhat different notations, the system describing the dynamics of *N* neurons (populations) is as follows

$$\begin{array}{c} {\dot{u}}_{i}=\frac{1}{\tau }(-u_{i}+\sum_{j=1}^{N}J_{ij}x_{j}-I-\lambda \sum_{j=1}^{N}x_{j}) \end{array}$$ (1)

where *u*_*j*_ is the activity variable (average membrane potential) of neuron *j*, *I* is a constant external input, *x*_*j*_ is the firing rate of neuron j, the coefficients *J*_*ij*_ express the strength of the excitatory connections from neuron *j* to neuron *i* and *τ* is the time constant, measured in miliseconds. The terms $-I-\lambda \sum_{j=1}^{N}x_{j}$ represent inhibition, discussed in more detail below. The firing rate is itself a monotonously increasing function of the activity variable with limiting values 0 and 1. This function is often taken as *x* = *g*(*u*) = (1 + *e*^−*u*/*μ*^)^−1^. In this work we will use an approximation of *g*, as shown below.

System (1) can be expressed in terms of firing rates by means of the transformation *x*_*i*_ = *g*(*u*_*i*_).

$$\begin{array}{c} {\dot{x}}_{i}=\frac{1}{\tau }x_{i}\left(1-x_{i}\right)(-\mu g^{-1}\left(x_{i}\right)-I-\lambda \sum_{j=1}^{N}x_{j}+\sum_{j=1}^{N}J_{ij}x_{j}),\;i=1,\ldots ,N. \end{array}$$

Learned patterns are steady state patterns of Eq (1) of the form (*ξ*_1_, …, *ξ*_*n*_), *ξ*_*j*_ = 0 or 1. In order to apply the linearized stability principle we need to be able to evaluate partial derivatives of the right hand side of Eq (1) at the learned patterns. However for such states the derivatives do not exist for the particular choice of *g*. This makes it impossible to apply the algebraic method of linearization (computation of eigenvalues of the linearized system) in the current models. We can remedy this using the approach introduced in [91] by replacing the function *g*^−1^(*x*) = ln *x* − ln(1 − *x*) by its Taylor expansion *f*_*q*_(*x*) at *x* = 1/2 up to some arbitrary order *q*. When we let *q* tend to infinity *f*_*q*_ tends uniformly to *g*^−1^ in any interval in (0, 1). In the following, for simplicity, we take the expansion to first order *f*_1_(*x*) = 4*x* − 2, (this corresponds to *q* = 1). A different choice of *q* would not significantly alter our results. After renaming the parameters we arrive at the equations

$$\begin{array}{c} {\dot{x}}_{i}=\frac{1}{\tau }x_{i}\left(1-x_{i}\right)(-\mu x_{i}-I-\lambda \sum_{j=1}^{N}x_{j}+\sum_{j=1}^{N}J_{ij}x_{j}),\;i=1,\ldots ,N \end{array}$$ (2)

The system (2) has the following fundamental property: any vertex, edge, face or hyperface of the cube [0, 1]^*N*^ is *flow-invariant*: trajectories with starting point in any one of these sets are entirely including in it. This implies in particular that the vertices are equilibria, or steady-states, of Eq (2). The vertices have coordinates 0 (inactive unit) or 1 (active unit). Hence vertices correspond to patterns for the neural network and whenever a vertex is a stable equilibrium it represents a learned pattern.

### The modelling of synaptic depression

According to the synaptic depression assumption the coefficients *J*_*ij*_ vary in time according to the rule:

$$\begin{array}{c} J_{ij}\left(t\right)=J_{ij}^{\text{max}}s_{j} \end{array}$$ (3)

where the evolution of the synaptic variable *s*_*i*_ is given as follows [102]

$$\begin{array}{c} {\dot{s}}_{i}=\frac{1-s_{i}}{\tau_{r}}-Ux_{i}s_{i} \end{array}$$ (4)

*τ*_*r*_ and *U* being the time constant of the recovery of the synapse and the maximal fraction of used synaptic resources.

### The modelling of excitatory connections

We assume that the matrix of excitatory connections (*J*^max^)_*i*, *j* = 1, …*n*_ is derived from a set of learned patterns which must be stable steady states of the system. Following [71] we use the Hebbian learning rule for sparse matrices, as introduced in [103], see also [104]. According to this rule the coefficients of the connectivity matrix (*J*^max^)_*i*, *j* = 1, …*n*_ (without synaptic depression) satisfy

$$\begin{array}{c} J_{ji}^{\text{max}}=\sum_{k=1}^{P}\frac{\left(\xi_{i}^{k}-p\right)\left(\xi_{j}^{k}-p\right)}{Np(1-p)}, \end{array}$$ (5)

where *ξ*^1^, …, *ξ*^*P*^ are the learned patterns, *N* is the total number of neurons and *p* is the ratio of active to inactive units, measuring the sparsity of the matrix *J*. Note that the matrix given by Eq (5) is symmetric.

Let us set *ν* = (*Np*(1 − *p*))^−1^. We simplify the expression (5) by introducing a change of variables and parameters, see also [104]. The rhs of Eq (2) can be rewritten as

$$\begin{array}{c} \frac{\nu }{\tau }x_{i}\left(1-x_{i}\right)(-\mu /\nu x_{i}-I/\nu -\lambda /\nu \sum_{j=1}^{N}x_{j}+\sum_{j=1}^{N}J_{ij}^{\max}s_{j}x_{j}) \end{array}$$

where now

$$\begin{array}{c} J_{ji}^{\text{max}}=\sum_{k=1}^{P}\left(\xi_{i}^{k}-p\right)\left(\xi_{j}^{k}-p\right) \end{array}$$ (6)

Remark that *J*^max^ is symmetric, while *J* at *t* > 0 will not be so (as long as some and not all neurons are active, see Eq (3)). In other words synaptic depression has the effect of breaking the symmetry of the connectivity matrix.

We rename parameters *μ*/*ν* as *μ*, etc., rescaling time by *t* = *t*′/*ν* and update the definition of *J*_*ij*_ in Eq (2) by $J_{ji}=J_{ji}^{\max}s_{j}$, with $J_{ji}^{\text{max}}$ given by Eq (6). Further, we assume that the connectivity matrix is sparse, which is consistent with neurophysiological data [105], as well as with computational models showing that a sparse matrix allows maximal storage capacity [106–110]. In the following we shall assume that *p* ≪ 1 (sparse matrix) and replace *p* by 0 in Eq (6). This guarantees that the weights $J_{ji}^{\text{max}}$ are positive, which is consistent with the assumption that they correspond to the excitatory connections. Moreover, given that *ν* is a constant between 0 and 1 and, due to the sparsity of $J^{\text{max}}$, is not particularly close to 0, we set, for simplicity, *ν* = *τ*. This choice does not qualitatively alter our results. Hence the context of our study is system (2) with *τ* = 1 and the goal is to find latching dynamics between learned patterns with the connectivity matrix given by Eq (6). As an intermediate stage of our investigation we will consider systems of the form (2) with weights that do not satisfy Eq (6).

### The modelling of inhibition

The term −*I* in Eq (2) corresponds to constant (tonic inhibition). Due to the presence of this term the pattern consisting of all neurons inactive is stable.

The term − λ∑*x*_*i*_ is the non-selective inhibition, depending on the activity of the specific neurons. This contribution should be thought of as feedback inhibition: a pyramidal neuron which is active excites some interneurons which contribute an inhibitory feedback. The choice of the dense inhibitory connectivity is supported by the experimentally known fact that interneurons are characterised by an extensive axonal arborisation, which allows each one of them to reach a large number of pyramidal cells in a local network [111].

### The modelling of noise

Noise plays a very important role of facilitating the transition from steady states that lose stability to the ones that follow in the sequence of latching dynamics. Therefore the noisy perturbation should not have the factor of *x*(1 − *x*), which would make it very small near the vertices, yet it must preserve the invariance of the cube [0, 1]^*N*^. We construct the noise term starting with white noise and subsequently modify it so that it points towards the interior of the cube. This noise term can be thought of as a fluctuation of the firing rate due to random presence or suppression of spikes. The role of the adjustment brought to white noise is to ensure that negative firing rates or firing rates greater than 1 do not arise. In practice, in our simulations we add a noisy perturbation to the initial condition at regular intervals of time, making sure that the perturbations are positive for firing rates near 0 and negative for firing rates near 1.

Sparse connectivity and the non-selective inhibition imt stable patterns contain only a few active neurons.

### Heteroclinic chains

One of the goals of this work is to show that latching dynamics is approximated by *heteroclinic chains*, which we introduce here. Given given a sequence of steady state patterns *ξ*^1^, …, *ξ*^*M*^, *M* < *P*, a *heteroclinic chain* consists of a sequence of connecting trajectories (dynamic transition patterns) between these patterns and of a sequence of time instances 0 < *t*_1_ < ⋯ < *t*_*M*_ such that the transition from *ξ*_*k*_ to *ξ*_*k*+1_ exists for the coefficients of the connectivity matrix *J*_*ij*_ evaluated at *t*_*k*_, that is $J_{ij}=J_{ij}^{\text{max}}s_{j}\left(t_{k}\right)$, where *i*, *j* = 1, 2, …, *n*.

We will show that latching dynamics is closely approximated by heteroclinic chains. Hence, finding a heteroclinic chain in our model becomes equivalent to the problem of finding heteroclini chains. This problem can therefore be formulated as follows:

**Problem**: let there be given a sequence of patterns *ξ*^1^, …, *ξ*^*M*^, *M* < *P*, where *ξ*^*k*^ and *ξ*^*k*+1^ share at least one active unit for all *k* = 1, …, *P* − 1. Under which conditions does there exist a sequence of connecting trajectories *ξ*^1^ → ⋯ → *ξ*^*P*^, so that a *heteroclinic chain* is realized between these patterns?

## Results

In this section we argue that latching dynamics is closely approximated by heteroclinic chains and subsequently investigate the possibility and feasibility of the existence of heteroclinic chains in our model. As justified in more detail below, sparse connectivity of excitation and dense connectivity of feedback inhibition imply that latching dynamics involves only a few learned patterns, consisting of a small number of active neurons. Moreover, as we argue below, there is significant overlap between the patterns. Hence we expect that there exists a small *subnetwork*, weakly connected to the rest of the network, which supports a heteroclinic chain. The connectivity matrix restricted to this subnetwork is not necessarily obtained from the learning rule (6). Based on this argument we break up the problem into two parts:

- we consider a small network (the prototype of a subnetwork), designing the connectivity matrix so that a heteroclinic chain connecting a priori specified patterns exists,
- we construct a larger network whose connectivity matrix is derived from the learning rule (6) such that the small network is its subnetwork. Our construction leads naturally to a matrix with sparse connectivity. It is known that connectivity in the brain is only about 10% [105, 112–116], hence our construction is consistent with the biophysical data.

We carry out this procedure for a few examples illustrating the general principle.

### Eigenvalue computations

The structure of Eq (2) makes the eigenvalues of the system linearized at each steady state pattern lying on a vertex of the hypercube [0, 1]^*N*^ easy to compute (diagonal Jacobian matrix). Let *ξ* = (*ξ*_1_, …, *ξ*_*N*_) be a vertex (hence *ξ*_*j*_ = 0 or 1), then the eigenvalue at *ξ* along the coordinate axis *x*_*k*_ has the form

$$\begin{array}{c} \sigma_{k}={\left(-1\right)}^{\xi_{k}}(-\mu \xi_{k}-I-\lambda \sum_{j=1}^{N}\xi_{j}+\sum_{j=1}^{N}J_{kj}^{\max}s_{j}\xi_{j}). \end{array}$$ (7)

The stability condition is now

(S) *σ*_*k*_ < 0 for all *k* = 1, 2, …, *n*.

Note that this algebraic method would not be available if we had not replaced the function *f*, equal to the inverse of the transfer function, by its Taylor polynomial.

The assumption of sparsity implies that for each *k* only a few $J_{kj}^{\max}$’s can be non-zero. This means that in a stable pattern only a few *ξ*_*j*_’s can be non-zero, otherwise the contribution of the non-selective inhibition would not allow the stability condition to hold.

Formula (7) and the stability condition (S) are the tools that will allow us to create conditions for the existence of heteroclinic chains and establish the role of the overlap between learned patterns. We show that such overlap is needed for the existence of a heteroclinic chain.

### Sparse coding and the relation between latching dynamics and heteroclinic chains

Consider a pattern *ξ* = (*ξ*_1_, *ξ*_2_, …, *ξ*_*N*_) and suppose *ξ* is a stable steady state of Eq (2) with *s*_*j*_ = 1, *j* = 1, …, *N*. We will argue that only a few of the components *ξ*_*j*_ can be significantly larger than 0. Assume this is not the case. Then, either there is a large number of *ξ*_*j*_’s satisfying 0 < *ξ*_*j*_ < 1 or there is a large number of *ξ*_*j*_’s equal to 1. In the first case λ∑*ξ*_*j*_ must be larger that ∑*J*_*ij*_ *ξ*_*j*_, so that the pattern cannot be a steady state. If the second case arises then the pattern can be a steady state but it cannot be stable, since the eigenvalues corresponding to the entries equal to 1 must be positive, see Eq (7). Hence the number of components that are not very close to 0 must be small. This property is an expression of sparse coding (few neurons code for every concept), which, in the context of our model, is a consequence of sparse connectivity in the excitatory network and dense connectivity of feedback inhibition.

The above argument implies that latching dynamics must necessarily take place near the surface of [0, 1]^*N*^, as the learned patterns have to be close to the surface and the transitions between them cannot be take a very long time, otherwise synaptic depression would weaken the excitatory connections making it unlikely for such transitions to occur. Hence the question is to identify dynamics on the boundary of the cube that could be a good approximation of latching dynamics. In fact the argument of sparse coding given above suggests that the dynamics is restricted to a subset of the boundary of the cube of relatively small dimension. In a heteroclinic chain we find a model of the dynamics in the edges of the cube which, when perturbed by noise gives a faithful representation of latching dynamics.

### Constructing heteroclinic chains

Due to the action of synaptic depression each of the learned patterns in a heteroclinic chain must lose stability due to one or more of the eigenvalues *σ*_*k*_ becoming positive. We assume that no two eigenvalues become positive at the same time, which implies that a noisy trajectory must follow the direction of the unstable eigenvalue. We will assume that in order to pass from one learned pattern to the next, the trajectory follows the edge corresponding to the unstable eigenvalue to the opposite vertex, which is a saddle point with a single unstable direction connecting to the next learned pattern in the chain. The chain will therefore be a sequence of *elementary chains* consisting of connections with three elements: a learned pattern *ξ*^*i*^ that becomes unstable due to synaptic depression (prime), a *transition* pattern ${\hat{\xi }}^{i}$ of saddle type, and the next learned pattern *ξ*^*i*+1^ (target). The active units in the pattern ${\hat{\xi }}^{i}$ correspond to the overlap between the patterns *ξ*^*i*^ and *ξ*^*i*+1^ The fact that the transition pattern should be unstable imposes another condition on the eigenvalues. These conditions will be presented in the next section.

We argue that an elementary chain is the most likely mechanism of transition from *ξ*^*i*^ to *ξ*^*i*+1^. It is certainly the simplest case dynamically. Any more complicated dynamics would be likely to increase the passage time, so that the target pattern could lose stability due to synaptic depression before becoming active in the chain. Finally, more complicated dynamics would require the existence of additional unstable eigenvalues leading to additional constraints on the matrix $J_{ij}^{\text{max}}$.

### Constraints on the connectivity matrix

We now state the algebraic constraints from the eigenvalue conditions (7) which define the parameter regions where heteroclinic chains could exist (see S1 File for a derivation of these conditions). These conditions are only necessary, in fact our numerics show that heteroclinic chains which follow a prescribed sequence of connections arise in a reliable manner in yet smaller parameter regions. The origin of these conditions is the requirement of stability of the steady states in the absence of synaptic depression combined with the requirement of the existence of a passage to the next steady state once the state currently attracting the dynamics loses stability due to synaptic depression. A cycle we consider joins a sequence of learned patterns *ξ*^1^ → ⋯ → *ξ*^*p*^ such that each of them has exactly *m* excited neurons (with entry 1) and the switching from one pattern to the next corresponds to switching the values in two entries. Possibly after re-arrangement of the indices it is no loss of generality to assume that

$$\begin{array}{c} \xi^{1}=\left(\overset{m\;times}{\overset{︷}{1,\ldots ,1}},0,\ldots ,0\right)\;,\;\xi^{2}=\left(0,\overset{m\;times}{\overset{︷}{1,\ldots ,1}},0,\ldots ,0\right),\ldots ,\xi^{p}=\left(0,\ldots 0,\overset{m\;times}{\overset{︷}{1,\ldots ,1}},0,\ldots ,0\right). \end{array}$$

In addition we have *p* − 1 transition patterns

$${\hat{\xi }}^{1}=\left(0,\overset{m-1\;times}{\overset{︷}{1,\ldots ,1}},0,\ldots ,0\right),\;{\hat{\xi }}^{2}=\left(0,\;0,\overset{m-1\;times}{\overset{︷}{1,\ldots ,1}},0,\ldots ,0\right),\ldots ,{\hat{\xi }}^{p-1}=\left(0,\ldots 0,\overset{m-1\;times}{\overset{︷}{1,\ldots ,1}},0,\ldots ,0\right).$$

We make a simplifying assumption that the entries of *J*^max^ are 0 outside of a band around the diagonal of width 2*m* − 1 (this is consistent with the requirement of the sparsity of the matrix). We introduce:

$$\begin{array}{c} \begin{array}{cc} & \Lambda_{i,k}=\sum_{l=0}^{m-1}J_{k,i+l},\;1\leq i\leq n-m+1,\;1\leq k\leq n\}\\ & \Lambda^{\text{max}}=\max_{i,k\notin \{i,\ldots i+m-1\}}\Lambda_{i,k}\\ & \Lambda^{\text{min}}=\min_{i,k\in \{i,\ldots i+m-1\}}\Lambda_{i,k}. \end{array} \end{array}$$ (8)

The requirement that the patterns *ξ*^1^, …*ξ*^*p*^ are stable in the absence of synaptic depression can be expressed, using Eq (7), by the condition

$$\begin{array}{c} m\lambda +I>\Lambda^{\text{max}} \end{array}$$ (9)

$$\begin{array}{c} m\lambda +I<\Lambda^{\text{min}}-\mu \end{array}$$ (10)

Other types of constraints come from the fact that, in the time interval of transition from one pattern to the next, the dynamics must approach a transition state from the direction of the prime pattern and leave in the direction of the target pattern. It means that there is a time instance ${\hat{t}}_{i}$ such that

$$\begin{array}{c} \sum_{j=i+1}^{m+i-1}J_{i,j}^{\max}s_{j}\left(t\right)<I+\left(m-1\right)\lambda <\sum_{j=i+1}^{m+i-1}J_{i+m,j}^{\max}s_{j}\left(t\right),\;t\in \left({\hat{t}}_{1}^{i},{\hat{t}}_{2}^{i}\right), \end{array}$$ (11)

which implies a weaker condition

$$\begin{array}{c} I+\left(m-1\right)\lambda <\min_{i}\Lambda_{i+m,i+1}. \end{array}$$ (12)

The combination of Eqs (9), (10) and (12) places severe restrictions on the parameters λ, *I* and $J_{ij}^{\text{max}}$. Additional constraints can be derived from the fact all the other directions of ${\hat{\xi }}_{i}$ have to be stable, in order to ensure the reliability of the cycle, but we did not explore these conditions here. For *m* = 2 we can use Eq (11) and the fact that there is only one synaptic variable pertaining to ${\hat{\xi }}^{i}$ to obtain the inequality: $J_{i,i+1}^{\max}<J_{i+2,i+1}^{\max}$. From the symmetry of the connectivity matrix we conclude that $J_{i,i+1}^{\max}<J_{i+1,i+2}^{\max}$. In other words, the elements on the upper diagonal and the lower diagonal must be increasing. This, combined with Eqs (9), (10) and (12), gives, for *m* = 2

$$\begin{array}{c} \begin{array}{cc} & \left(i\right)\;J_{i,i+1}^{\max}<J_{i+1,i+2}^{\max},\;i=1,\ldots n-1\;\text{(upper}\;\text{diagonal}\;\text{elements}\;\text{are}\;\text{increasing)}\\ & \left(\text{ii}\right)\;I+\lambda <J_{32}^{\max}\\ & \left(\text{iii}\right)\;I+2\lambda >J_{p,p+1}^{\max}\\ & \left(\text{iv}\right)\;I+2\lambda <\min_{i=1,\ldots ,p}\left(J_{i,i}^{\max}+J_{i,i+1}^{\max}\right)\\ & \left(v\right)\;I+2\lambda <\min_{i=2,\ldots ,p+1}\left(J_{i,i}^{\max}+J_{i,i-1}^{\max}\right). \end{array} \end{array}$$ (13)

The property of increasing diagonal elements, in practice, prevents the existence of long chains as the large coefficients will activate the corresponding neurons just due to the presence of noise.

We use Eq (13) to select the parameters for our numerical examples. For the details of the derivation of the algebraic constraints we refer to S1 File.

### Conditions based on slow/fast dynamics

To take advantage of the fact that the synaptic variables *s*_*i*_ are slow compared to the firing rates we write the equation for the *s*_*i*_’s in the form

$$\begin{array}{c} {\dot{s}}_{i}=\varepsilon \left(\left(1-s_{i}\right)-\rho x_{i}s_{i}\right), \end{array}$$ (14)

with

$$\begin{array}{c} \rho =\tau_{r}U\;\text{and}\;\varepsilon =\frac{1}{\tau_{r}}. \end{array}$$

In this formulation the model has a time scale separation and *ρ* is a regular parameter.

The transitions $\xi^{i}\to {\hat{\xi }}^{i}\to \xi^{i+1}$ are governed by Eq (2), which implies that as *ε* → 0 each one of them lasts for an approximately constant positive amount of time. It follows that as *ε* → 0 the change in *s*_*i*_ in the transition period tends to 0, that is *s*_*i*_ remains close to a constant value, approximated by bifurcation points, corresponding to the loss of stability of *ξ*_*i*_. These values can be derived in a recurrent manner as follows:

1. At *t* = 0 *s*_1_ = *s*_2_ = 1. Until the loss of stability of *ξ*_1_ it holds that *x*_1_ ≈ 1 and *x*_2_ ≈ 1. Hence, in that period, we set: *x*_1_ = *x*_2_ = 1 and Eq (14) becomes
  $$\begin{array}{c} {\dot{s}}_{i}=\varepsilon \left(-\left(1+\rho \right)s_{i}+1\right), \end{array}$$ (15)
  where *i* = 1, 2. Since *s*_1_ and *s*_2_ have the same initial condition they remain equal. Using Eq (7) we derive that time when *ξ*_1_ loses stability is defined by:
  $$\begin{array}{c} I+\mu +2\lambda I=J_{11}s_{1}\left(t_{B}\right)+J_{12}s_{2}\left(t_{B}\right). \end{array}$$
  Hence the bifurcation is given by the *s*_1_ and *s*_2_ values:
  $$\begin{array}{c} s_{1}=s_{1}^{B,1}=\frac{I+\mu +2\lambda }{J_{11}+J_{12}},\;s_{2}=s_{2}^{B,2}=\frac{I+\mu +2\lambda }{J_{11}+J_{12}}. \end{array}$$
2. Assuming that *s*_2_ does not change during the transition $\xi^{1}\to {\hat{\xi }}^{1}\to \xi^{2}$, at the beginning of the period when the trajectory is near *ξ*_2_, we have $s_{2}=s_{1}^{B,2}$ and *s*_3_ = 1. The evolution of (*s*_2_, *s*_3_) until the next stability loss is Eq (15) with *i* = 2, 3. Note that Eq (15) is a linear equation and hence can be solved:
  $$\begin{array}{c} \begin{array}{cc} & s_{2}\left(t\right)=e^{-\varepsilon (1+\rho t)}\left(s_{1}^{B,2}-1\right)+\frac{1}{\rho +1}\\ & s_{3}\left(t\right)=e^{-\varepsilon (1+\rho t)}\frac{\rho }{1+\rho }+\frac{1}{1+\rho }. \end{array} \end{array}$$ (16)
  By Eq (7) we must now choose *t*_*B*_ so that
  $$\begin{array}{c} I+\mu +2\lambda =J_{22}s_{2}\left(t_{B}\right)+J_{23}s_{3}\left(t_{B}\right). \end{array}$$
  Hence the values of (*s*_2_, *s*_3_) corresponding to the point of the loss of stability of *ξ*^2^ are $s_{2}^{B,1}=s_{2}\left(t_{B}\right)$ and $s_{2}^{B,2}=s_{3}\left(t_{B}\right)$. To obtain $s_{2}^{B,1}$ and $s_{2}^{B,2}$ we solve the linear system
  $$\begin{array}{c} (\begin{array}{ccc} -(s_{1}^{B,1}-\frac{1}{1+\rho }) & 1 & 0\\ -\frac{\rho }{1+\rho } & 0 & 1\\ 0 & J_{22} & J_{23} \end{array})(\begin{array}{c} F_{B},\\ s_{2}^{B,1}\\ s_{2}^{B,2} \end{array})=(\begin{array}{c} \frac{1}{1+\rho }\\ \frac{1}{1+\rho }\\ I+\mu +2\lambda \end{array}). \end{array}$$ (17)
  with $F_{B}=e^{-\varepsilon \left(1+\rho \right)t_{B}}$. The values of $s_{2}^{B,1}$ and $s_{2}^{B,2}$ can now be found by solving this linear equation (the determinant of the matrix is easily shown to be non-zero).
3. The process of the derivation of the bifurcation values is iterative. If $s_{k-1}^{B,2}$ is known then $s_{k}^{B,1}$ and $s_{k}^{B,2}$ are obtained by solving the linear equation
  $$\begin{array}{c} (\begin{array}{ccc} -(s_{k-1}^{B,1}-\frac{1}{1+\rho }) & 1 & 0\\ -\frac{\rho }{1+\rho } & 0 & 1\\ 0 & J_{kk} & J_{kk+1} \end{array})(\begin{array}{c} F_{B},\\ s_{k}^{B,1}\\ s_{k}^{B,2} \end{array})=(\begin{array}{c} \frac{1}{1+\rho }\\ \frac{1}{1+\rho }\\ I+\mu +2\lambda \end{array}). \end{array}$$ (18)
  This calculation allows us to check for a given matrix *J*, if the specified heteroclinic chain exists for sufficiently small *ε*. The first set of conditions is
  $$\begin{array}{c} 1>s_{i}^{B,j}>\frac{1}{1+\rho }\;i=1,\ldots ,P,\;j=1,2. \end{array}$$ (19)
  The second set of conditions is obtained based on the requirement that, for each *i* = 1, 2, …, *P*, at the *i*th bifurcation point the following properties hold:
  - *ξ*^*i*+1^ must be stable,
  - ${\hat{\xi }}^{i}$ must be a saddle with the direction of *e*_*i*_ stable (*e*_*i*_ is the vector with *i*th component 1 and the other components 0),
  - *ξ*^*i*^ must be stable in the direction of *e*_*i*+1_.

Explicit conditions can be derived in an iterative manner using Eq (7), in the sequel we do this numerically in the context of specific examples. If these conditions are satisfied then a chain exists for sufficiently small *ε* and small noise. If the conditions are violated then the chain does not exist unless the noise is large and the transitions are driven exclusively by noise.

### Satisfying the learning rule

Our results show that, given a small network, the parameters have to be tuned quite precisely to obtain a heteroclinic chain. Adding the requirement that the matrix is obtained using the learning rule (6) gives an even more severe constraint. We have constructed examples of networks supporting heteroclinic chains with each neuron involved in some of the patterns forming the chain. In each case the connectivity coefficients we used had larger values than given by the patterns involved in the chain alone. To solve this problem we designed a method of defining a larger system, with the connectivity matrix of the form

$$\begin{array}{c} {\tilde{J}}^{\text{max}}=(\begin{array}{cc} J^{\text{max}} & A\\ A^{T} & B \end{array}) \end{array}$$ (20)

and added learned patterns which do not participate in the chain but with an overlap with the patterns forming the chain, so that the matrix $\tilde{J}$ is obtained using the learning rule (6). The matrix *A* consists of many blocks with few non-zero coefficient that are small in comparison to the entries of $J^{\text{max}}$. The matrix *B* is block diagonal, with the off-diagonal entries in each of the blocks equal to 1. The added learned patterns must satisfy the constraints (9) and (10) to ensure their stability. This way the matrix is sparse (about 25% non-zero elements in the example we constructed). There is no natural algorithm to construct ${\tilde{J}}^{\text{max}}$, so we refrain from making any further specifications. We constructed ${\tilde{J}}^{\text{max}}$ for a specific example, see S2 File.

### A simple example—An elementary chain

In this section we examine the simplest case of a network of three neurons and two learned patterns

$$\begin{array}{c} \xi^{1}=\left(1,1,0\right)\;\text{and}\;\xi^{2}=\left(0,1,1\right). \end{array}$$ (21)

This example is the prototype of an elementary chain. In this section we describe the simplest case of constructing a heteroclinic chain, namely a sequence of connecting orbits (dynamic transition patterns) along edges of the cube in $R^{3}$ connect these patterns in a chain. This requires the presence of one intermediate equilibrium ${\hat{\xi }}^{1}=\left(0,1,0\right)$, which by assumption is not a learned pattern and has an unstable direction along the coordinate which passes from 0 to 1 in the sequence

$$\begin{array}{c} \xi^{1}\to {\hat{\xi }}^{1}\to \xi^{2}. \end{array}$$ (22)

Computing the connectivity matrix from the learning rule (6) with patterns *ξ*^1^ and *ξ*^2^ is straightforward and gives

$$\begin{array}{c} J^{\text{max}}=(\begin{array}{ccc} 1 & 1 & 0\\ 1 & 2 & 1\\ 0 & 1 & 1 \end{array}) \end{array}$$ (23)

Applying formula (7) with *N* = 3 and *J*^max^ given by Eq (23) it is easily checked that the two learned patterns have negative eigenvalues, hence are stable, in the absence of synaptic depression, iff 1 < *I* + 2λ < 2 − *μ*. This is our first requirement.

In the remaining of this section $\sigma_{k}^{i}$, resp. ${\hat{\sigma }}_{k}$, will denote the eigenvalue at *ξ*_*i*_, resp. $\hat{\xi }$ along *x*_*k*_.

We now “switch on” synaptic depression. As time elapses, synaptic weights will be modified according to Eq (4). For a given pattern (steady-state of Eq (2)) on a vertex of the cube [0, 1]^3^) the evolution of the synaptic variable *s*_*i*_ (*i* = 1, 2 or 3) can be of two types: (i) if *x*_*i*_ = 0 the value *s*_*i*_ = 1 is a steady-state of Eq (4); (ii) if *x*_*i*_ = 1 then *s*_*i*_ decreases monotonically towards the limit value *S* = (1 + *τU*)^−1^ which is the steady-state of Eq (4) at *x*_*i*_ = 1.

Let us describe in terms of the eigenvalues associated with each pattern, the scenario which would lead to the expected heteroclinic chain.

First, the weakening of the synaptic variable *s*_1_ should modify the stability of the learned pattern *ξ*_1_ in such a way that a trajectory will appear connecting *ξ*_1_ to the intermediate $\hat{\xi }$ along the first coordinate *x*_1_. This entails that the eigenvalue $\sigma_{1}^{1}$ at *ξ*_1_ (eigenvalue along coordinate *x*_1_), which initially is negative, becomes positive after some time. This is a kind of *dynamic bifurcation*, in which time plays the role of a bifurcation parameter through the evolution of the synaptic variables. Simultaneously we want the eigenvalue ${\hat{\sigma }}_{1}$ at $\hat{\xi }$ become negative at finite time so that this state is attracting along direction *x*_1_. By Eq (7) a sufficient condition for this is $-I-\lambda +J_{12}^{\max}S<0$.

The second step is to check conditions which would allow for the existence of a trajectory connecting $\hat{\xi }$ to *ξ*_2_ along the edge with coordinate *x*_3_. This requires that the eigenvalue ${\hat{\sigma }}_{3}$ be positive after some time (possibly already at *t* = 0), hence by Eq (7), $-I-\lambda +J_{32}^{\max}S>0$. The two conditions we just derived are incompatible with matrix Eq (23), so we can conclude that this matrix does not admit the heteroclinic chain Eq (22).

From the above discussion we can infer that a necessary condition for the existence of a chain Eq (22) is that $J_{32}^{\max}>J_{12}^{\max}$. Since *J*^max^ is symmetric this means that its upper diagonal must have strictly increasing coefficients. In addition to these conditions we also request that: (i) *ξ*_1_ be “more” stable in the *x*_2_ direction than in the *x*_1_ direction, i.e. $\sigma_{2}^{1}<\sigma_{1}^{1}<0$ at *t* = 0, so that a trajectory starting close to this equilibrium will first destabilize in the *x*_1_ direction (it may of course not destabilize at all); (ii) the *x*_2_ direction at $\hat{\xi }$ is stable; (iii) *ξ*_2_ is stable when *t* large enough. This imposes additional conditions on the coefficients of *J*^max^. Let us show that the matrix

$$\begin{array}{c} J^{\text{max}}=(\begin{array}{ccc} 2 & 1 & 0\\ 1 & 3 & 2\\ 0 & 2 & 2 \end{array}) \end{array}$$ (24)

satisfies all these conditions and hence admits the heteroclinic chain Eq (22). Of course this is an ad’hoc construction, however we shall show in S2 File that Eq (24) is a submatrix of the connectivity matrix of a subnetwork of a large sparse network under the learning rule (6) (see Eq (20)).

For Eq (24) the eigenvalues computed using Eq (7) are as follows:

$$\begin{array}{c} \begin{array}{cc} \sigma_{1}^{1} & =I+2\lambda -(2s_{1}+s_{2}-\mu )\\ \sigma_{2}^{1} & =I+2\lambda -(s_{1}+3s_{2}-\mu )\\ \sigma_{3}^{1} & =-I-2\lambda +2s_{2}\\ {\hat{\sigma }}_{1} & =-I-\lambda +s_{2}\\ {\hat{\sigma }}_{2} & =I+\lambda -(3-\mu )\\ {\hat{\sigma }}_{3} & =-I-\lambda +2s_{2}\\ \sigma_{1}^{2} & =-I-2\lambda +s_{2}\\ \sigma_{2}^{2} & =I+2\lambda -(3s_{2}+2s_{3}-\mu )\\ \sigma_{3}^{2} & =I+2\lambda -(2s_{2}+2s_{3}-\mu ). \end{array} \end{array}$$

From this we obtain the following conditions:

1. 2 < *I* + 2λ < 3 (stability of the learned patterns in absence of synaptic depression),
2. 3*S* < *I* + 2λ ($\sigma_{1}^{1}$ becomes >0 in finite time),
3. 1 < *I* + λ (${\hat{\sigma }}_{1}<0$),
4. *I* + λ < 2*S* (${\hat{\sigma }}_{3}$ becomes >0 in finite time),
5. *I* + 2λ < 2*S* + 2 ($\sigma_{3}^{2}<0$).

**Fig 1:**
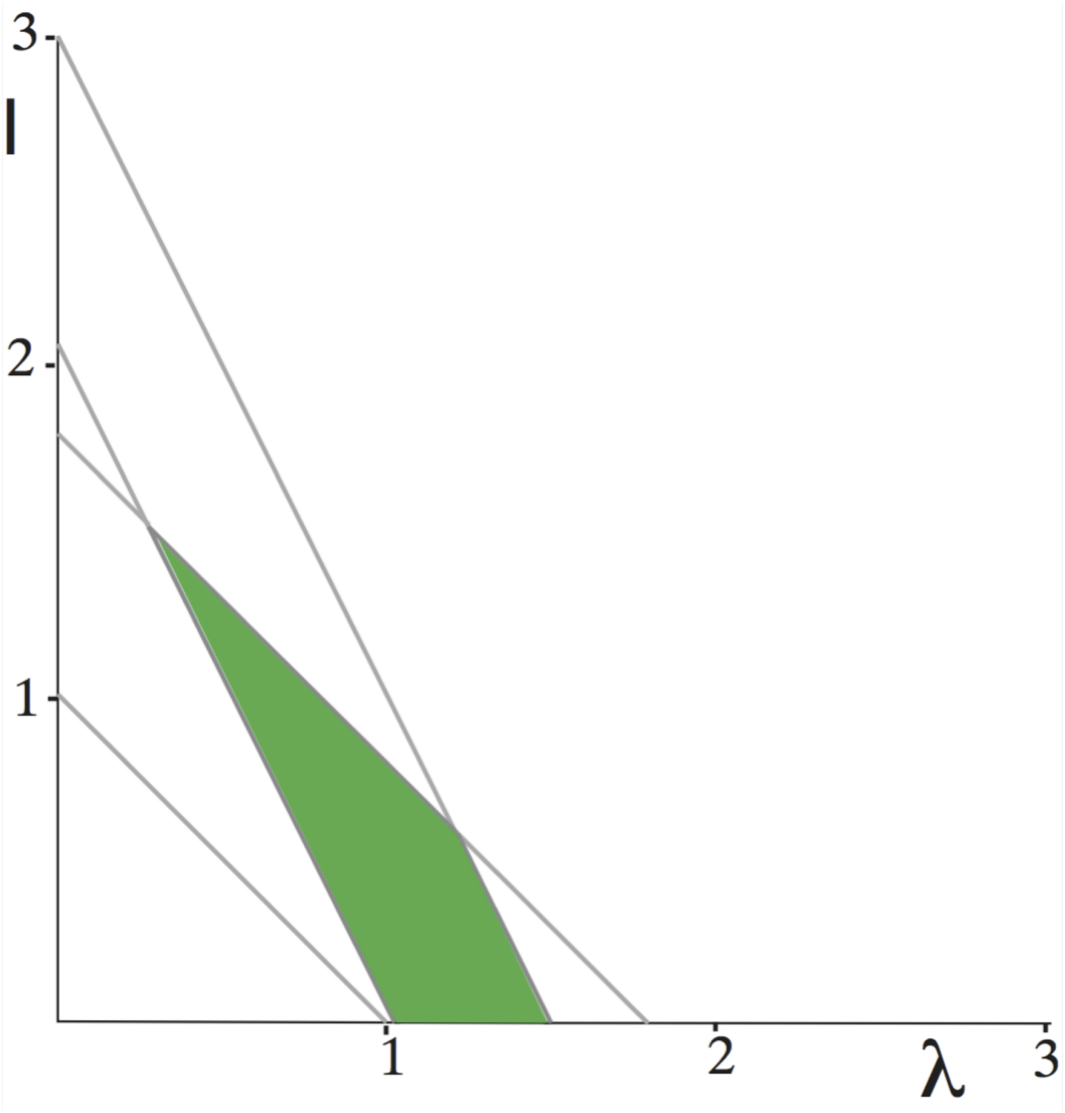
The domain of values (λ, *I*) which allow for existence of a heteroclinic dynamics with matrix Eq (24) and *S* = 1/1.4.

These conditions are all satisfied if *τ* and *U* are chosen such that 2/3 < *S* and the point (λ, *I*) lies in the triangle bounded by the lines *I* > 0, 2 < *I* + 2λ and *I* + λ < 2*S*. The value of *S* = (1 + *τU*)^−1^ being given, these conditions can be conveniently represented graphically. Fig 1 shows an example with *τ* = 100 and *U* = 0.004.

**Fig 2:**
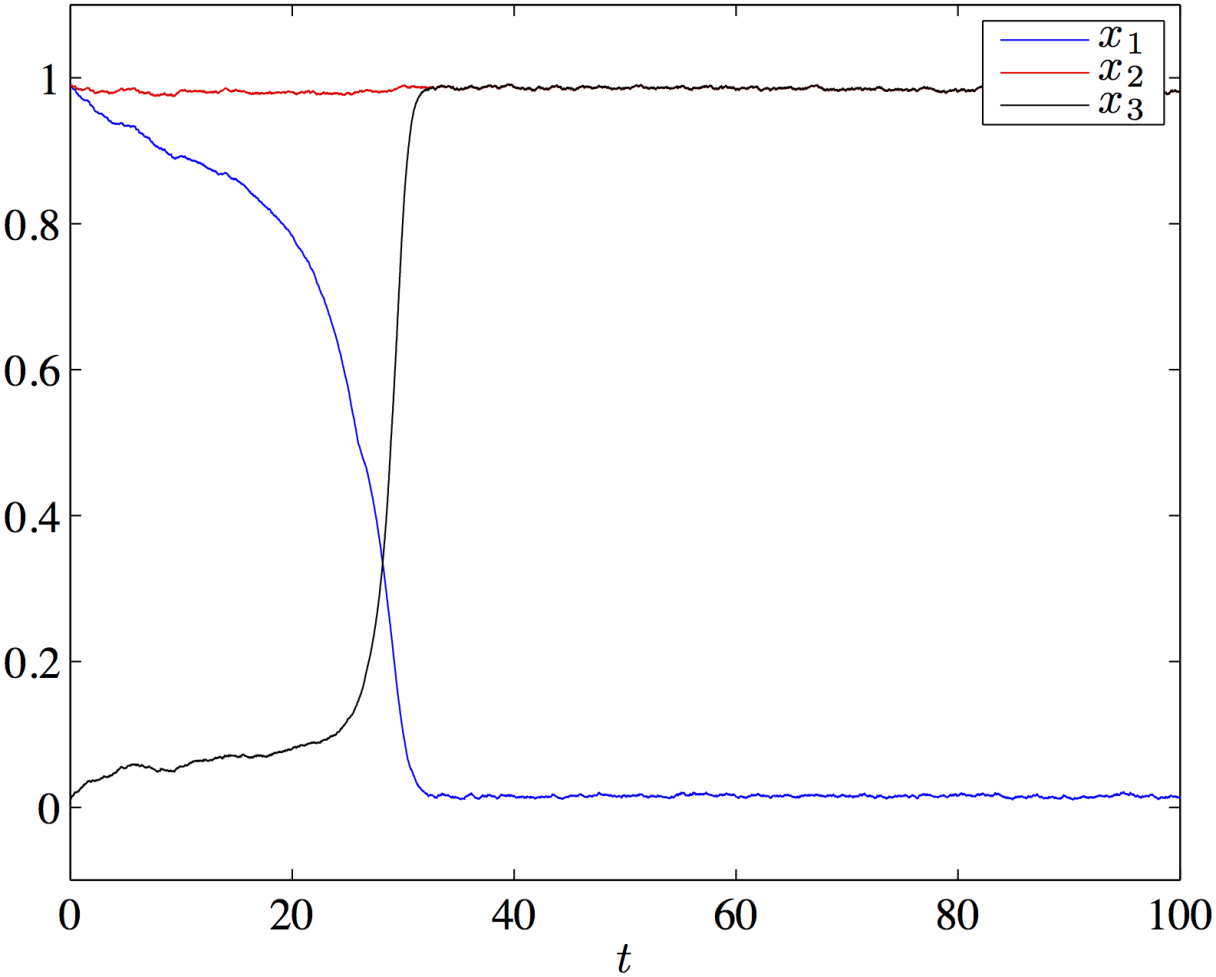
Panel (a): time series of *x*_1_ (blue), *x*_2_ (red) and *x*_3_ (green). Panel (b): time series of *s*_1_ (blue), *s*_2_ (red) and *s*_3_ (green).

Let us illustrate numerically this result. We have integrated the Eqs (2) and (4) with *N* = 3 and coefficient values *τ* = 100, *U* = 0.004, *I* = 0.15 and λ = 1.2. Fig 2 shows a time series of *x*_*I*_(*t*) (upper figure) and *s*_*i*_(*t*) (lower figure) with an initial condition starting close to *ξ*_1_. In order to observe the transitions Eq (22) in reasonable time we have incorporated a noise in the code, in the form of a small random deviation from initial condition on the *x*_*i*_ variables at each new integration time (simulation with Matlab).

## Numerical examples

### A case with five neurons and the extended network of 61 neurons

**Fig 3:**
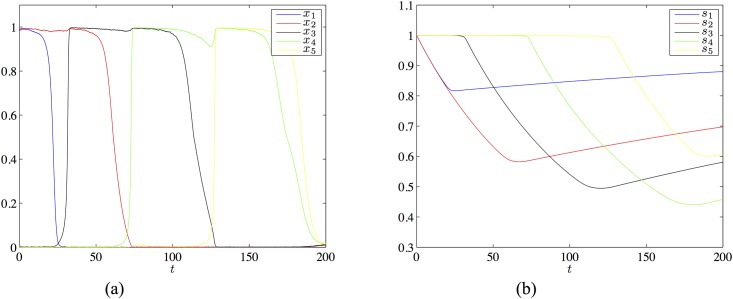
The chain of four patterns in the network of 5 neurons with the transition matrix Eq (25) and the parameters *I* = 0.3, λ = 3.4, *μ* = 3.1, *τ_r_* = 400 and *U* = 0.01. Panel (a) shows the evolution of the firing rate variables, panel (b) shows the evolutions of the synaptic variables.

We consider

$$\begin{array}{c} J^{\text{max}}=(\begin{array}{ccccc} 9 & 3 & 0 & 0 & 0\\ 3 & 10 & 5 & 0 & 0\\ 0 & 5 & 11 & 6 & 0\\ 0 & 0 & 6 & 11 & 7\\ 0 & 0 & 0 & 7 & 11 \end{array}), \end{array}$$ (25)

with *I* = 0.3, λ = 3.4, *μ* = 3.1, *τ*_*r*_ = 400 and *U* = 0.01. This matrix and these parameter values meet all conditions for the existence of a heteroclinic chain joining the patterns *ξ*^1^ = (1, 1, 0, 0, 0), …, *ξ*^4^ = (0, 0, 0, 1, 1), however Eq (25) does is not derived from the learning rule (6). In Fig 3 we show a simulation of a chain of four states existing for the above parameters.

**Fig 4:**
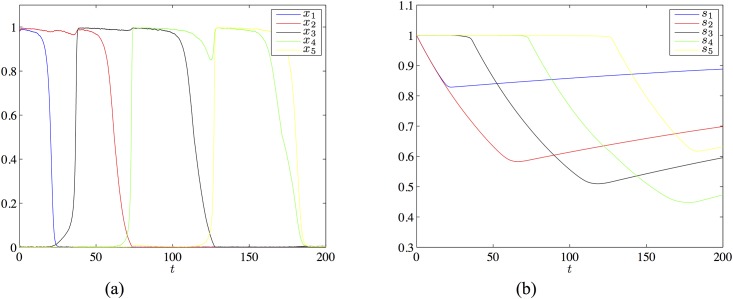
The chain of four patterns in the network of 61 neurons obtained by extending the five neuron example with the transition matrix Eq (25). The parameter settings are as the same as in the simulation of Fig 3. Panel (a) shows the evolution of the firing rate variables, panel (b) shows the evolutions of the synaptic variables.

We extended this network to a network of 61 neurons with the connectivity matrix satisfying the learning rule (6), using the approach described earlier. The details of the construction can be found in S2 File. Fig 4 shows a simulation for the required chain joining the states whose first five components are ass *ξ*^1^ = (1, 1, 0, 0, 0), …, *ξ*^4^ = (0, 0, 0, 1, 1) specified above and the remaining 56 components are 0.

#### Sparsity of the extended network

Electrophysiological studies suggest that connectivity in the brain is sparse with only approximately 10% of pairs of neurons connected [105, 112–116]. In the extended network obtained in this section and in S2 File the ‘emptiness’ of the matrix (fraction of zero-weight synapses) is above 75%, which is consistent with neurophysiological data as well as with computational models showing that a sparse matrix allows maximal storage capacity [106–110]. The present results show that sparsity is necessary not only to improve storage capacity (ensure the stability of learned patterns) but also to enable the sequential activation of patterns. Indeed, in the case of Hebbian learning considered here, heteroclinic chains involving patterns defined by the activity of neurons e.g. 1-6 are possible only if the synaptic matrix obeys conditions on the efficacies along the subdiagonal. These conditions depend in turn on the role of additional neurons (n) among a large number of ‘non-coding neurons’ taken into account in the learning equation. This is possible under conditions of sparse coding of the patterns.

#### Reliability of the chain

**Fig 5:**
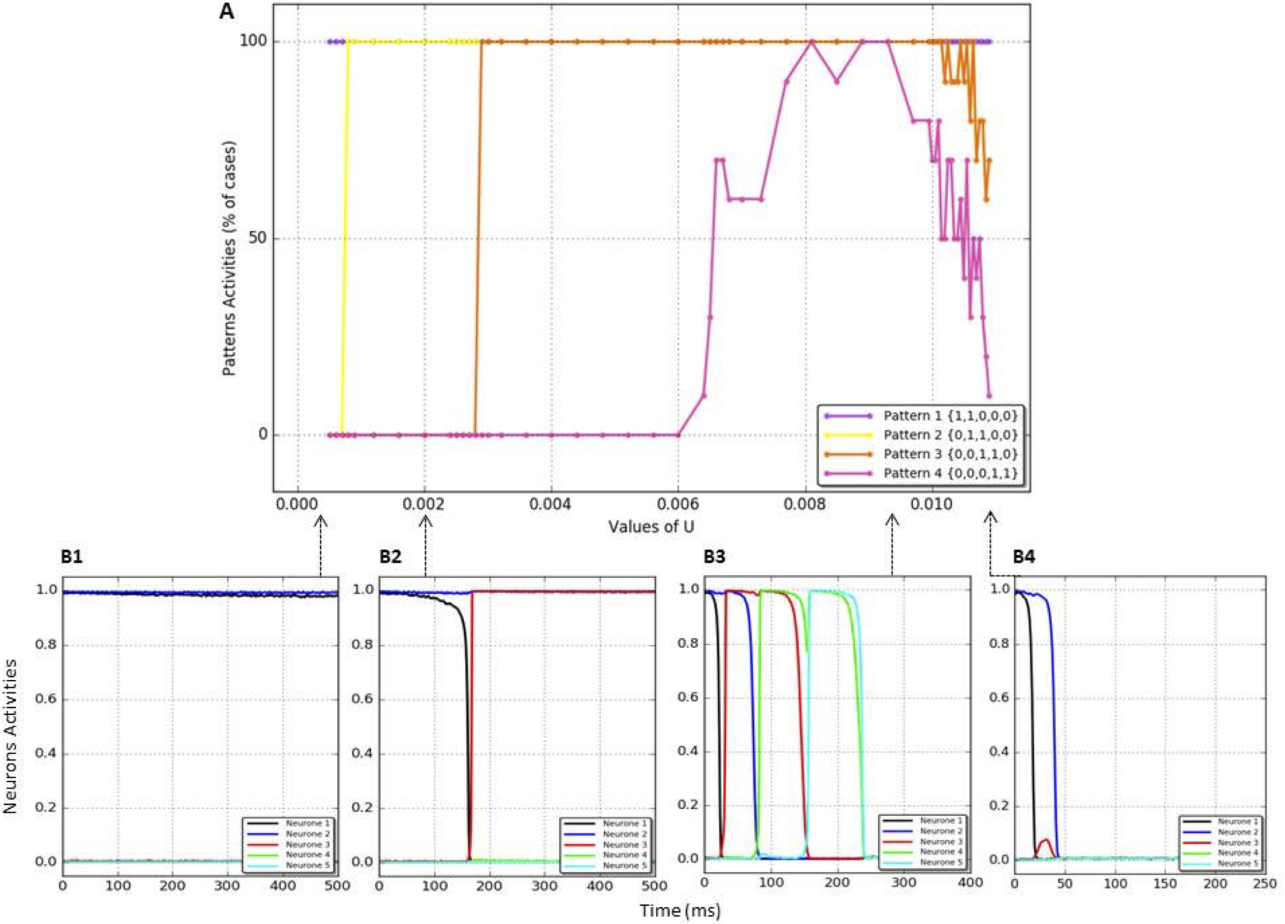
Panel A. Reliability of the chain of four patterns in the network of 61 neurons as a function of U. Panels B1-4. Activities of neurons coding for the patterns in the chain as a function of time for four representative values of U.

We have computed the reliability of the chain with the prescribed sequence (1, 1, 0, 0, 0) → (0, 1, 1, 0, 0) → (0, 0, 1, 1, 0) → (0, 0, 0, 1, 1) in the extended network of 61 neurons, with respect to the parameter *U* (maximal fraction of used synaptic resources). For parameter values distributed in the interval 0.0005 ≤ *U* ≤ 0.110 we performed 10 simulations with the same initial conditions for each of the chosen parameter values. Fig 5 shows the distribution, for different values of U, of the proportion of the simulations for which a given pattern was activated in the chain.

Panel A of Fig 5 shows that the activation of the full length chain (all of the four successive patterns) is possible for a limited range of values of U (from .0081 to .0093, see panel B3). Even within this range of values of U and with fixed parameter values, the chain does not always fully develop on every simulation due to the presence of noise. Further, for values of U lower or higher than this range, the chain does not fully develop for two different reasons. On the one hand, when U decreases, the length of the activated chain decreases because the network stays in the state corresponding to the first pattern (panel B1) or to the second pattern with slow transition time (panel B2). On the other hand, when U increases, the length of the activated chain decreases because the network does not stay in the state corresponding to the first but does not activate the second pattern either, ending in a state where no neuron is activated (panel B4). Taken as a whole, these results show that the value of U determines the reliability of the chain in terms of number of patterns activated, with the patterns occurring later in the chain less likely to be activated. Further, the value of U also determines the state of the network after the first pattern, ranging from this first pattern or the different following patterns (low values of U) to an absence of activity of the neurons (high values of U). For the optimal range of values of U, the reliability of the chain is maximal but not perfect due to the presence of noise.

#### Slower synaptic depression leads to more reliable chains

**Fig 6:**
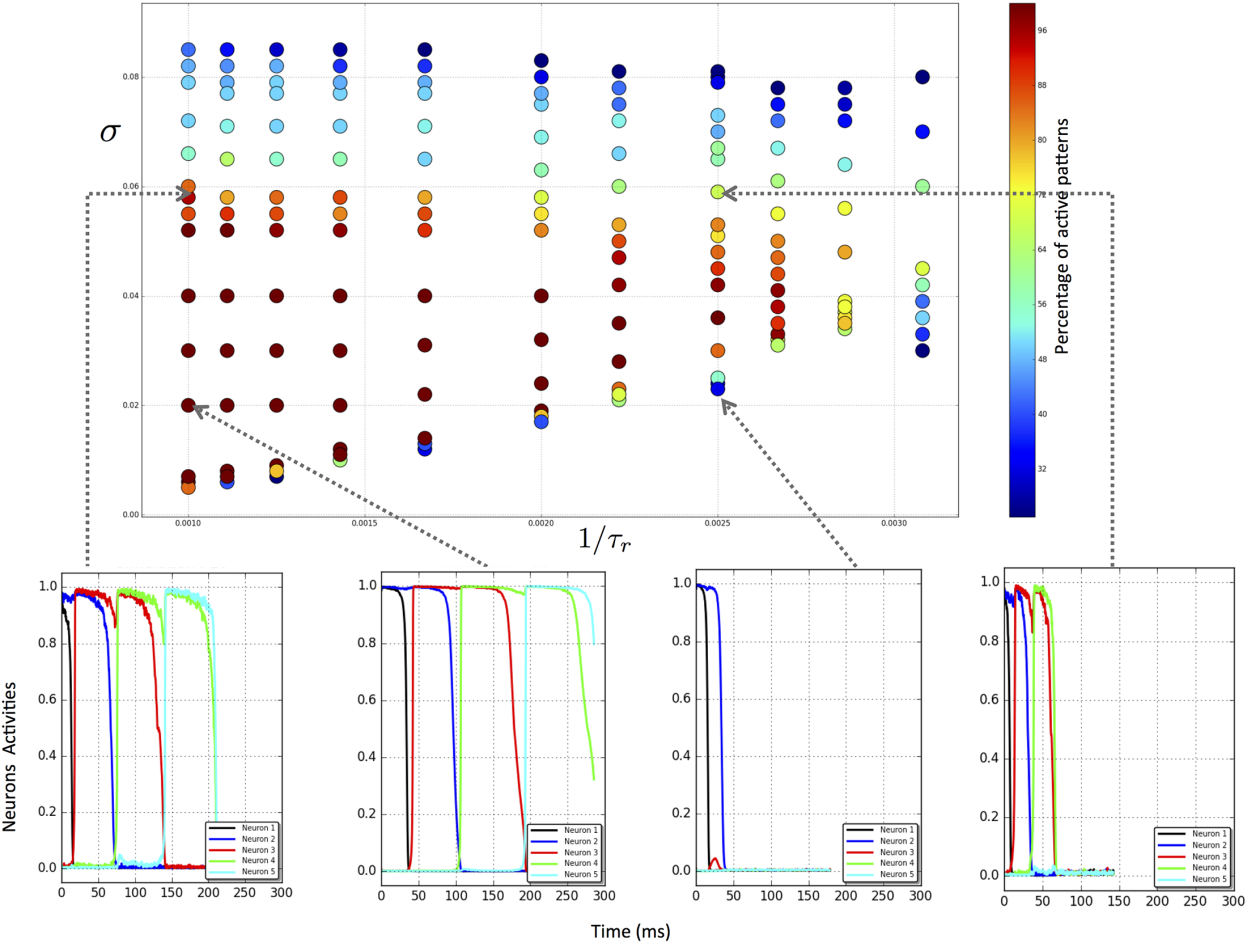
Activation of heteroclinic chains as a function of the time scale separation *ε* = 1/*τ_r_* and the amplitude of the noise *σ*. The top panel shows the simulations marked by dots in the (*ε*, *σ*) plane, colour coded according to the extent and reliability of the activation of the heteroclinic chain, as indicated by the colour bar on the right. Lower panels show sample time traces corresponding to a selection of the parameter points, as indicated.

Here we show some simulation complementing our analysis of the effect of varying the speed of the evolution of the synaptic variables *s*_*j*_. Earlier we introduced new parameters $\varepsilon =\frac{1}{\tau_{r}}$ and *ρ* = *τ*_*r*_*U* and studied the effect of decreasing *ε*, while keeping *ρ* fixed, which is equivalent to simultaneously increasing *τ*_*r*_ and decreasing *U*. We showed that this has the effect of decreasing the threshold of the noise amplitude needed for activation of a heteroclinic chain. This result is confirmed by simulations, as shown in Fig 6. In addition, our simulations show that the curve marking the upper limit of the noise window for which heteroclinic chains are activated also increases with the decrease of *ε*. Altogether, the window of noise for which the chains are activated is larger if the synaptic variable evolves more slowly. Our hypothesis, based on the simulations shown in Fig 6, is that time scale separation increases the reliability of the deterministic fast component of the dynamics, limiting the role of the stochastic component to the initiation of the transitions from one state to the next.

### A chain connecting six patterns with *m* = 2

In this section we present an example of a longer chain involving six neurons and 5 patterns. We do not construct the extension to a large network with a matrix derived from the learning rule (6). This would be possible using the approach outlined earlier and carried out in detail for the example of five neurons in S2 File. However the matrix we consider has larger entries than the one of the preceding section (five neurons), which implies that a larger extended network would be needed.

We consider the following connectivity matrix:

$$\begin{array}{c} J^{\text{max}}=(\begin{array}{cccccc} 13 & 6 & 0 & 0 & 0 & 0\\ 6 & 14 & 13 & 0 & 0 & 0\\ 0 & 13 & 16 & 14 & 0 & 0\\ 0 & 0 & 14 & 20 & 15 & 0\\ 0 & 0 & 0 & 15 & 20 & 16\\ 0 & 0 & 0 & 0 & 16 & 20 \end{array}) \end{array}$$ (26)

**Fig 7:**
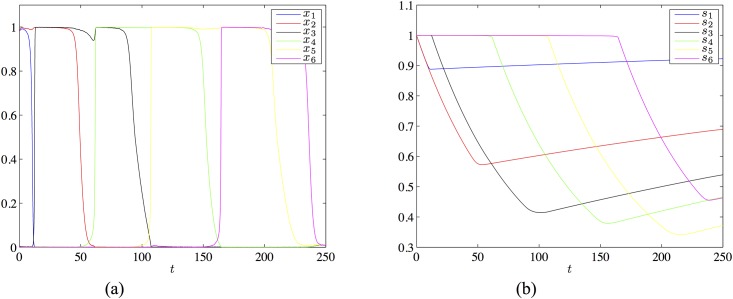
Chain of five patterns with 6 neurons and *m* = 2. Panel (a) shows the firing rates *x_j_* and panel (b) shows the synaptic variables *s_j_*. Color code: blue = *x*_1_, red = *x*_2_, black = *x*_3_, green = *x*_4_, cyan = *x*_5_, magenta = *x*_6_. Same code for *s_j_*.

This matrix satisfies the necessary conditions eq-condsmt based on the learned patterns *ξ*^1^ = (1, 1, 0, 0, 0, 0), *ξ*^2^ = (0, 1, 1, 0, 0, 0), *ξ*^3^ = (0, 0, 1, 1, 0, 0), *ξ*^4^ = (0, 0, 0, 1, 1, 0) and *ξ*^5^ = (0, 0, 0, 0, 1, 1). For the choice of parameters *I* = 0.48, λ = 8, *μ* = 1.2, *τ*_*r*_ = 600 and *U* = 0.012 and for a range of noise amplitudes this matrix gives the following heteroclinic chain/latching dynamics:

Starting with initial condition close to *ξ*^1^, the dynamics visits successively *ξ*^2^, …, *ξ*^5^, the transition form *ξ*^*i*^ to *ξ*^*i*+1^ passing through the intermediate (not learned) state ${\hat{\xi }}^{i}$ with only one excited neuron at rank *i* + 1, see Fig 7. Observe that as long as a variable *x*_*j*_ is “large” (close to 1) the corresponding synaptic variable *s*_*j*_ decreases until *x*_*j*_ comes close to 0. Then *s*_*j*_ increases, according to the time evolution driven by Eq (4).

### A case of a shared neuron with m = 3

This example gives a different option for the neural coding of items. Thus far the principle of our model has been that each item (e.g. the prime or target) is coded by a pattern of activity of all neurons in the network. As a consequence only one item can be ‘activated’ at a given time in a heteroclinic chain. The simplest case is when two patterns are activated in succession: the pattern coding for the prime followed by a pattern coding for the target [70, 72–74]. In this case either the prime or the target is ‘activated’ at a given time. Such priming mechanism can account for neuronal activities recorded in nonhuman primates in priming protocols where a prime is related to a single target. In that case priming relies on the successive activation of the presented prime and of the predicted target [15, 69]. However, in human studies priming is reported not only for targets directly related to the prime (Step 1 targets), but also for targets indirectly related to the prime through a sequence of one (Step 2 targets) or two (Step 3 targets) intermediate associates of the prime that are activated after the prime and before the target (e.g. [25] for a review). Such indirect priming has been accounted for by network models in which Step 1, Step 2 and Step 3 associates to the primes were coded by neural populations that can be activated simultaneously [25]. The present model of heteroclinic chains is of particular interest to account for the sequential activation of items involved in step priming. However, in priming studies the prime can still be reported by participants after processing of the target [117]. This suggests that the prime must be available in working memory at the end of the activation of the sequence of Step associates, that is neurons coding for the prime must be active at the end of the heteroclinic chain. The possibility to activated the prime in after several associates have been activated in a chain is no reproduced by models of priming based on latching dynamics [70, 71]. In the present model, a way to for neurons coding for the prime to be actvated at the end of the heteroclinic chain is simply to consider that a pattern (attractor state) does not correspond to a single item, but rather corresponds to several items each corresponding to the activity of a subgroup of neurons. In that case the activity of a neuron would correspond to the average activity of a population of neurons coding for and item [22]. Such population coding is consistent with recent models of priming in the cerebral cortex [25, 40, 118]. Intuitively, the first pattern in the chain codes for the prime only while the next pattern 2 in the chain codes for the combination of the prime and of the Step 1 target together, the pattern 3 codes for the Prime, Step1 target and Step 2 target, and so on. This way the population coding for the prime would be active throughout the entire computation.

In this section we present an example of a system of five neurons with three active neurons in each pattern and one neuron present in each pattern. For this we use the following connectivity matrix:

$$\begin{array}{c} J^{\text{max}}=(\begin{array}{ccccc} 12 & 2 & 4 & 4 & 4\\ 2 & 6 & 3 & 0 & 0\\ 4 & 3 & 6 & 4 & 0\\ 4 & 0 & 4 & 7 & 6\\ 4 & 0 & 0 & 6 & 7 \end{array}) \end{array}$$ (27)

**Fig 8:**
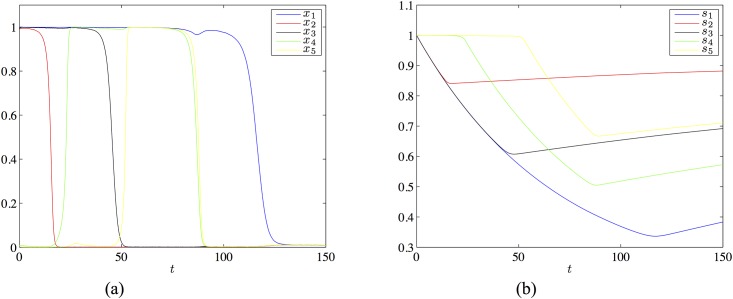
Chain of three patterns with 5 neurons, m = 3 and one shared neuron. Panel (a) shows the firing rates *x_j_* and panel (b) shows the synaptic variables *s_j_* Color code: blue = *x*_1_, red = *x*_2_, black = *x*_3_, green = *x*_4_, cyan = *x*_5_. Same code for *s_j_*.

*τ*_*r*_ = 400, *x*_max_ = 1, *U* = 0.012. For this system, for the choice of the parameters *I* = 0.5, λ = 2.8 and *μ* = 1 we find by simulation a chain joining *ξ*_1_ = (1, 1, 1, 0, 0), *ξ*_2_ = (1, 0, 1, 1, 0), *ξ*_3_ = (1, 0, 0, 1, 1), ${\hat{\xi }}_{1}=\left(0,1,1,0,0\right)$ and ${\hat{\xi }}_{2}=\left(0,0,1,1,0\right)$. The time series of the solution is shown in Fig 8.

The above analysis of the network behavior shows that heteroclinic chains can develop in the case where a neuron (i.e. a population coding for the prime) is active for all the successive patterns in the chain. In other words the overlap between populations coding for different items is such that a subgroup of neurons coding for an item (e.g. the prime) can remain activated while another subgroup can be deactivated as the chain progresses. The network is able to keep previous stimuli activated (e.g. a prime) while at the same time it can activate a sequence of items (i.e. associates to the prime) that can be predicted on the basis of the prime. The compatibility between changing patterns in the chain and stable activity of a neuron or population of neurons allows to account for two fundamental properties exhibited by the brain, within a unified model. Due to the structure of the overlap in the coding of the memory items, this example combines the population coding used classically in models of priming in the cerebral cortex, in which a given item is coded by a given population of neurons, and the distributed coding used in Hopfield types models of priming, in which a given item is coded by the pattern of activity of all neurons in the network. In this way the present model aims at unifying our understanding of the coding of items in memory and of priming processes between these items.

## Discussion

The present study provides the first analysis of the sufficient conditions for heteroclinic chains of overlapping patterns in the case of a symmetric Hebbian learning rule. Heteroclinic chains closely approximate *latching dynamics*, hence they are good candidates to account for priming processes reported in human and nonhuman primates. Here priming-based prediction is seen as the activation—by a pattern presented to the network—of a pattern not (yet) presented. Within this framework, heteroclinic chains account for the activation or inhibition of neurons so that the network codes for the ‘target’ pattern before its actual presentation, under conditions of overlap with the pattern coding for the ‘prime’ pattern. Heteroclinic chains account for different dynamics of activity of neurons reported in nonhuman primates during the delay between the prime and target: some neurons active for the prime and for the target remain active (pair coding neurons [1, 2]), some neurons active for the prime but not for the target are deactivated and some neurons not active for the prime but active for the target exhibit an increased activity during the delay, which corresponds to prospective activity [11–15]. The model of latching dynamics has been adapted to allow for the existence of heteroclinic chains, by replacing the equation for the membrane potential by the equation for the firing rate, with the nonlinearity replaced by its polynomial approximation (to arbitrary order), so that the dynamics is well defined even when the firing rate takes its minimal (0) or maximal (1) values [91]. In the modified model we were able to identify some of the restrictions imposed on the network by the requirement of the existence of heteroclinic chains. From a modelling perspective, this is a step in bridging the gap between Hopfield-type models of priming and cortical network models of priming. The present model provides a mathematically tractable description of the reliability of sequences of patterns used to model priming in non-human primates and in human. Itl exhibits latching dynamics reported to account for priming processes and their perturbations, and it calculates spike rates of neurons coding for items in terms of overlap between related populations. It could serve future applications and to better understand perturbation of priming processes reported in pathologies of priming, such as Alzheimer disease or schizophrenia, by analyzing the reliability of sequences as a function of network parameters usually considered as subtending perturbations of priming (noise, dopaminergic activity, synaptic connectivity); [25, 119–121].

It should be noted that recent work by [122] has shown that short term memory can be induced as persistent activity in clustered networks without synaptic learning. This effect called *cluster reverberation*, could be the main mechanism by which short-term memory (sensory or working memory) works in the brain. In this model learned patterns are by nature metastable and latching dynamics can arise as activation of sequences of patterns. We expect that our approach could also apply to analyse the dynamics in cases of clusters of neurons.

### Predictions on neural activities in priming

The present model makes predictions regarding the possibility for the prime to remain activated (remembered) or not (forgotten) as the chain progresses. In the network, activating patterns coding for several Step 1, Step 2, etc targets would make difficult the simultaneous persistent activity of the prime, due to retroactive interference based on inhibition generated by the ‘step’ targets [40]. The corresponding experimental prediction that could be tested in priming experiments is that the activity of neurons coding for the prime would decrease when successive targets are predicted in memory even though they are not actually presented. This could be visible in nonhuman primates on a decrease of the retrospective activity of neurons coding for the prime when a series of ‘Step’ targets is predicted. The behavioral counterpart in humans would be a decrease in the reportability of the prime when the length of the sequence of targets to predict increases.

### Asymmetry of priming and of synaptic efficacies

Brunel [106] recently pointed out the possibility that the optimal synaptic matrix depends on the constraint imposed on the network, either storing patterns as stable states or storing patterns to be activated in sequences. The present results show that heteroclinic chains are possible with symmetric matrices built through Hebbian learning and specify the necessary conditions for sequences to arise in the network. Although asymmetric connections can improve the ability of the network to activate sequences of patterns, they are not a necessary condition. However, the present results also show that the conditions for heteroclinic chains impose strong constraints on the structure of the synaptic matrix, suggesting that although symmetric weights can be optimal for storage capacity, they are not an optimal solution for the activation of sequences. If the network codes for a given pattern 1 at a given time, the activation of the next pattern 2 in a chain requires that two given neurons *i* and *j* activate each other or not depending on their state within each pattern. For example, *i* but not *j* can be activated in 1, and the opposite in 2. Hence for an optimal sequence 1 → 2, *i* should activate *j* but *j* should not activate *i*. The present results show that the symmetry of the weights can be compensated by the sparsity of the network at the expense of an increasing number of neurons necessary to code for the patterns. Even though asymmetric heteroclinic chains are possible with symmetric synaptic efficacies, further analysis of heteroclinic chains with asymmetric learning rules would bring new evidence on the specific role of asymmetric weights on the required level of sparsity and on the reliability of latching dynamics.

## Supporting information

S1 File Constraints on the connectivity matrix. (PDF)

S2 File Constructing a sparse network which satisfies the learning rule (6) and supports a heteroclinic chain. (PDF)
