# Supplementary material for: Latching dynamics in neural networks with synaptic depression
Source: PLoS One. 2017 Aug 28;12(8):e0183710. doi: 10.1371/journal.pone.0183710 (PMC5573234; doi:10.1371/journal.pone.0183710)
Supplement: S1 File — (PDF) [file pone.0183710.s001.pdf]

S 1 Constraints on the connectivity matrix: derivation of the algebraic constraints

Stability of learned patterns in the absence of synaptic depression

The example with three neurons ($n = 3$) shown earlier corresponds to the ideal situation where there is an open domain in the space of parameters of the problem, such that the heteroclinic chain connecting learned patterns along edges of the hypercube $[0, 1]^n$ indeed exists. Ideally we would like to derive a set of necessary and sufficient conditions in a general setting, however this seems to be much more difficult to obtain with larger networks and longer chains. Nevertheless dynamics following edges of the hypercube that visit learned patterns in an a priori specified sequence, with the coefficients derived using the conditions presented in Results section, can be observed as evidenced by our simulations. Here we derive numerous conditions that the coefficients of $J^{\max}$ must satisfy, which strongly restrict the choice of these quantities.

Recall that, in our setting, a learned pattern is a stable (when no synaptic depression is present) vertex equilibrium $\xi = (\xi_1, \dots, \xi_n)$ where each $\xi_j = 0$ or 1. Let us consider a sequence of learned patterns $\xi^1 \to \cdots \to \xi^p$ such that each of them has exactly m excited neurons (with entry 1) and the switching from one pattern to the next corresponds to switching values in two entries. Possibly after re-arrangement of the indices it is no loss of generality to assume that

$$\xi^1 = (\overbrace{1, \dots, 1}^{m\ times}, 0, \dots, 0)\ ,\ \xi^2 = (0, \overbrace{1, \dots, 1}^{m\ times}, 0, \dots, 0), \dots, \xi^p = (0, \dots 0, \overbrace{1, \dots, 1}^{m\ times}, 0, \dots, 0).$$

For any pattern ξ^i ($i < p$) in the above sequence, let y be the coordinate corresponding to its first non-zero entry and z be the coordinate corresponding to the first 0 entry after the sequence of 1's. For example in ξ^1, $y = x_1$ and $z = x_{m+1}$. We also note $\hat{\xi}^i$ the vertex equilibrium which makes the connection from ξ^i to ξ^{i+1}: its coordinates are those of ξ^i except the i-th entry which is 0 instead of 1. By assumption these intermediate states $\hat{\xi}^i$ are not learned patterns and are saddles for the dynamics of (2) with all s_i fixed to 1. We specifically require the eigenvalue at $\hat{\xi}^i$ along y be negative and the eigenvalue along z be positive.

Initially the learned patterns ξ^i are stable equilibria of eqs (2) and synaptic variables s_i are assigned their maximal value 1. As time evolves the values $s_j(t)$ corresponding to excited neurons ($x_j(t) > 0$) decrease and the eigenvalues (7) are modified. We expect that after some time t_i the eigendirection y at ξ^i becomes unstable and a heteroclinic connection is established along this edge to $\hat{\xi}^i$. The synaptic variables play the role of dynamically evolving parameters. If they did not depend on time, the situation could be described as a classical bifurcation: as long as the eigenvalues at ξ^i and $\hat{\xi}^i$ are both negative an unstable equilibrium exists on the edge joining the two states. When the synaptic variables associated with the excited neurons decrease this equilibrium moves towards ξ^i and when it merges in it, the heteroclinic connection is established (see figure S1). We also expect $\hat{\xi}^i$ to have an unstable eigendirection along z so that a heteroclinic connection exists from $\hat{\xi}^i$ to ξ^{i+1}. These scenarios can be explicitely described by writing the equations restricted to the edges with variable coordinates y and z.

This process should repeat itself from $i = 1$ to $i = p - 1$.

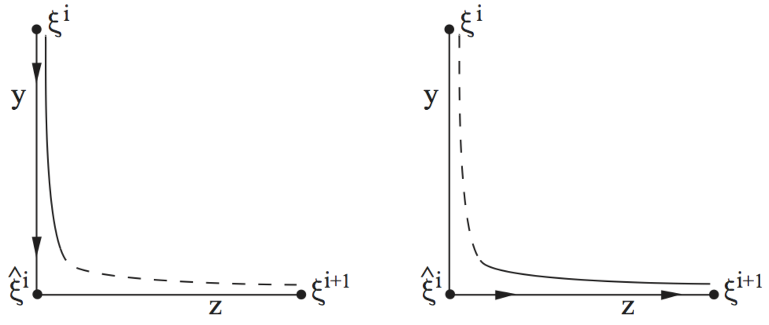

Fig. S1: Connections from ξ^i to ξ^{i+1} through $\hat{\xi}^i$. Left: $t < t_i$, right: $t > t_i$.

We make a simplifying assumption that the entries of $J^{\max}$ are 0 outside of a band around the diagonal of width $2m-1$. It follows from this assumption and from (7) that when $s_j = 1$ the eigenvalues at each pattern ξ^i have the following form

$$\begin{aligned} \sigma_k^i &= -I - m\lambda + \sum_{j=i}^{i+m-1} J_{k,j}^{\max} \text{ when } k < i \text{ or } k > i+m-1 \\ \sigma_k^i &= \mu + I + m\lambda - \sum_{j=i}^{i+m-1} J_{k,j}^{\max} \text{ when } i \leq k \leq i+m-1 \end{aligned} \tag{S2}$$

We require all these eigenvalues to be negative. This gives two conditions, which we now specify. Let

$$\Lambda_{i,k} = \sum_{l=0}^{m-1} J_{k,i+l}, \quad 1 \leq i \leq n-m+1, \quad 1 \leq k \leq n\}.$$

We define

$$\Lambda^{\max} = \max_{i,k \notin \{i,\ldots i+m-1\}} \Lambda_{i,k}, \quad \Lambda^{\min} = \min_{i,k \in \{i,\ldots i+m-1\}} \Lambda_{i,k}.$$

The two requirements become

$$m\lambda + I > \Lambda^M \tag{S3}$$

$$m\lambda + I < \Lambda^m - \mu \tag{S4}$$

Note that $\Lambda_{i,k} \neq 0$ if $|k-i| < m$ and equals 0 otherwise. Note also that the computation of Λ^m involves non-diagonal elements only. Hence by making the diagonal elements large enough, it is possible to ensure that conditions (S3) and (S4) hold.

Necessary conditions on $J^{\max}$ for the transition from one pattern to the next

We analyze the transition at $\hat{\xi}^i$ between ξ^i and ξ^{i+1}. Let $\sigma^i_y, \hat{\sigma}^i_z$ be the eigenvalues along eigendirections y and z at $\hat{\xi}^i$ and time t. From (7) we have

$$\begin{aligned}\hat{\sigma}^i_y &= -I - (m-1)\lambda + \sum_{j=i+1}^{m+i-1} J^{\max}_{i,j} s_j(t) \\ \hat{\sigma}^i_z &= -I - (m-1)\lambda + \sum_{j=i+1}^{m+i-1} J^{\max}_{i+m,j} s_j(t).\end{aligned} \tag{S5}$$

We want that during an interval of time $0 < \hat{t}^i_1 < t < \hat{t}^i_2$ $\hat{\sigma}^i_y$ becomes negative while $\hat{\sigma}^i_z$ is positive, which give the condition

$$\sum_{j=i+1}^{m+i-1} J^{\max}_{i,j} s_j(t) < I + (m-1)\lambda < \sum_{j=i+1}^{m+i-1} J^{\max}_{i+m,j} s_j(t),\ t \in (\hat{t}^i_1, \hat{t}^i_2) \tag{S6}$$

The quantity $S = (1 + \tau U)^{-1}$ is the minimal value that can be attained by the synaptic variables s_j. Recall the assumption that the elements of $J^{\max}$ are 0 outside a strip of width $2m-1$ about the diagonal. Then (S6) implies a weaker but simpler condition:

$$S \max_i \ \Lambda_{i,i+1} < I + (m-1)\lambda < \min_i \Lambda_{i+m,i+1}. \tag{S7}$$

When $m = 2$ (S6) implies the following simple condition:

Proposition 1. *If $m = 2$, then (S6) implies that $J^{\max}_{i,i+1} < J^{\max}_{i+2,i+1} = J^{\max}_{i+1,i+2}$ (by symmetry of the matrix). Therefore the upper diagonal of $J^{\max}$ has coefficients with increasing values (and the subdiagonal too).*

Indeed, this condition does not hold for matrix (23) while matrix (24) satisfies it.
For the case $m = 2$, in addition to the requirement that the off diagonal coefficients must increase, we deduce the following simple conditions:

$$\begin{aligned}&\text{(i) } J^{\max}_{i,i+1} < J^{\max}_{i+1,i+2},\ i = 1, \dots n-1 \text{ (upper diagonal elements are increasing)} \\ &\text{(ii) } S J^{\max}_{p,p+1} < I + \lambda < J^{\max}_{32} \\ &\text{(iii) } I + 2\lambda > J^{\max}_{p,p+1} \\ &\text{(iv) } I + 2\lambda < \min_{i=1,\dots,p} (J^{\max}_{i,i} + J^{\max}_{i,i+1}) \\ &\text{(v) } I + 2\lambda < \min_{i=2,\dots,p+1} (J^{\max}_{i,i} + J^{\max}_{i,i-1}).\end{aligned} \tag{S8}$$

A geometric representation of (S8) similar to Fig. 1 could be constructed. It is straightforward to verify that the conditions are satisfied for the coefficients of (26).
