# Supplementary material for: Latching dynamics in neural networks with synaptic depression
Source: PLoS One. 2017 Aug 28;12(8):e0183710. doi: 10.1371/journal.pone.0183710 (PMC5573234; doi:10.1371/journal.pone.0183710)
Supplement: S2 File — (PDF) [file pone.0183710.s002.pdf]

S 1 Constructing a sparse network which satisfies the learning rule (6) and supports a heteroclinic chain.

A straightforward calculation shows that none of the matrices $J^{\max}$ considered in the previous sections are obtained using the learning rule (6) from the patterns $\xi^1, \ldots, \xi^p$. It is natural to ask if these matrices can be derived using formula by adding more neurons and more learned patterns, so that the patterns $\xi^1, \ldots, \xi^p$ forming the heteroclinic chain are a part of a larger ensemble of learned patterns and the extended connectivity matrix has the form Our fundamental assumption (postulate) throughout this work is that a learned pattern must be stable in the absence of synaptic depression. In this section we show that the answer is positive in two cases we have investigated: the case with three neurons and $J^{\max}$ as in Results Section, and a more involved case with five neurons and four patterns in the heteroclinic chain. We suspect these results can be extended to more general situations but this is yet to be proved.

The case with $n = 3$

Note that the condition $J^{\max}_{32} > J^{\max}_{21}$ derived in Results Section would imply that there must exist a learned pattern different than ξ^2, in which neurons 2 and 3 are active and neuron 1 is not. This is obviously not possible with three neurons. In this section we show that it is possible to obtain a connectivity matrix in a network of six neurons that is derived using the learning rule (6) and has the matrix (24) in the top left corner. We write

$$\xi^1 = (1, 1, 0, 0, 0, 0) \text{ and } \xi^2 = (0, 1, 1, 0, 0, 0).$$

These are the patterns ξ^1 and ξ^2 generalized to the network of six neurons. In addition we consider the patterns:

$$\xi^3 = (0, 1, 1, 1, 0, 0), \quad \xi^4 = (1, 0, 0, 0, 0, 1), \quad \xi^5 = (0, 0, 0, 1, 1, 0), \quad \xi^6 = (0, 0, 0, 0, 1, 1). \quad \text{(S1)}$$

Note that ξ^3 satisfies precisely the condition stated above, i.e. neurons 2 and 3 are active and neuron 1 is not. The purpose of adding pattern ξ^4 is to make $J^{\max}_{11} = 2$. The role of patterns ξ_5 and ξ_6 is to ensure the stability of the added patterns as steady states of (2) without synaptic depression.

One can easily verify that the matrix

$$J^{\max} = \begin{pmatrix} 2 & 1 & 0 & 0 & 0 & 1 \\ 1 & 3 & 2 & 1 & 0 & 0 \\ 0 & 2 & 2 & 1 & 0 & 0 \\ 0 & 1 & 1 & 2 & 1 & 0 \\ 0 & 0 & 0 & 1 & 2 & 1 \\ 1 & 0 & 0 & 0 & 1 & 2 \end{pmatrix} \quad \text{(S2)}$$

is obtained by using formula (6) from the patterns ξ^1, ξ^2, ξ^3, ξ^4, ξ^5 and ξ^6. In addition we verify that the learned patterns ξ^1, ξ^2, ξ^3, ξ^4, ξ^5 and ξ^6 are stable steady states of (2) without synaptic depression,

ie where J_{ij} have been replaced by $J_{ij}^{\max}$. We perform the computation for the pattern ξ^3 and show that the additional condition $3\lambda + I < 4$ is needed. Clearly there exist choices of λ and I so that this condition as well as conditions 1.-5. stated in Results Section all hold.
Proceeding analogously as in Results Section we obtain the following eigenvalues:

$$\begin{aligned}
\sigma_1^3 &= -I - 3\lambda + s_2,\ \sigma_2^3 = I + 3\lambda - (3s_2 + 2s_3 + s_4 - \mu),\ \sigma_3^3 = I + 3\lambda - (2s_2 + 2s_3 + s_4 - \mu),\\
\sigma_4^3 &= I + 3\lambda - (s_2 + s_3 + 2s_4 - \mu),\ \sigma_5^3 = -I - 3\lambda + s_4,\ \sigma_6^3 = -I - 3\lambda
\end{aligned}$$

These expressions are all negative for $s_2 = s_3 = s_4 = 1$ and $\mu = 0$ if $1 < 3\lambda + I < 4$. The calculations for the other patterns are similar to the calculations in Results Section and lead to the condition $2 < 2\lambda + I < 3$, i.e. condition 1.

A case with five neurons

Recall the example of five neurons supporting a chain of four elements occurring for the connectivity matrix

$$J^{\max} = \begin{pmatrix} 9 & 3 & 0 & 0 & 0 \\ 3 & 10 & 5 & 0 & 0 \\ 0 & 5 & 11 & 6 & 0 \\ 0 & 0 & 6 & 11 & 7 \\ 0 & 0 & 0 & 7 & 11 \end{pmatrix}, \tag{S3}$$

with $I = 0.3$, $\lambda = 3.4$, $\mu = 3.1$, $\tau_r = 400$ and $U = 0.01$. This matrix and these parameter values meet all conditions for the existence of a heteroclinic chain joining the patterns $\xi^1 = (1, 1, 0, 0, 0), \ldots, \xi^4 = (0, 0, 0, 1, 1)$, however (S3) does not follow from learning rule (6). In figure 3 we show a simulation of a chain of four states existing for the above parameters.

Our goal is to extend it to a matrix satisfying the specification of the learning rule (6), based on a set of stable equilibrium patterns containing the ones that appear in the chain. Our approach is as in the example with $n = 3$ treated earlier. The existence of such an extension was announced in Results Section, here we carry out the construction in detail.

We consider a network with 61 neurons, with patterns of activity represented by vectors with 61 components. which we introduce below. We first introduce some notation: let $e_j \in \mathbb{R}^{61}$ be the vector with 1 in the jth spot and 0's elsewhere. Similarly, $e_{j,k}$ is the vector with 1's in the jth and kth spots and 0's elsewhere, $e_{j,k,l}$ the vector with 1's in the jth, kth and lth spots and 0's elsewhere, etc.

The extended network has the four learned patterns to be joined by a chain, which extend the patterns $\xi^1, \ldots, \xi^4$:

$$e_{1,2}, \quad e_{2,3}, \quad e_{3,4} \quad \text{and} \quad e_{4,5}. \tag{S4}$$

In addition it has the patterns of the form

$$
\begin{aligned}
&e_{1,2,j},\ j=6,7\ , \quad e_{2,3,j},\ j=20,21,22,23\\
&e_{3,4,j},\ j=34,35,36,37,38\ , \quad e_{4,5,j},\ j=48,49,50,51,52,53\\
&e_{1,j},\ j=6,7\ , \quad e_{1,7,j},\ j=20,21,22,23\\
&e_{2,j},\ j=6,7,8,9\ , \quad e_{3,j},\ j=20,21,22,23\\
&e_{4,j},\ j=34,35,36\ , \quad e_{5,j},\ j=48,49,\ldots,57\ .
\end{aligned}
\tag{S5}
$$

and the patterns of the form

$$
\begin{aligned}
&e_{i,j},\ i=6,7,\ldots,19,\ j=6,7,\ldots,19,\\
&e_{i,j},\ i=20,21,\ldots,33,\ j=20,21,\ldots,33,\\
&e_{i,j},\ i=34,35,\ldots,47,\ j=34,35,\ldots,47,\\
&e_{i,j},\ i=48,49,\ldots,61,\ j=48,49,\ldots,61.
\end{aligned}
\tag{S6}
$$

The role of patterns (S5) is to strengthen the weights in $J^{\max}$. The patterns (S6) play the role of stabilizing the patterns (S5).

The matrix derived from all the patterns by applying the learning rule eq:hebb rule is:

$$
\tilde{J}^{\max} = \begin{pmatrix}
J^{\max} & A_1 & A_2 & A_3 & A_4\\
A_1^T & L_1 & M & 0_{14\times 14} & 0_{14\times 14}\\
A_2^T & M^T & L_2 & 0_{14\times 14} & 0_{14\times 14}\\
A_3^T & 0_{14\times 14} & 0_{14\times 14} & L_3 & 0_{14\times 14}\\
A_4^T & 0_{14\times 14} & 0_{14\times 14} & 0_{14\times 14} & L_4
\end{pmatrix},
\tag{S7}
$$

where A_1, A_2, A_3 and A_4 are 14×5 matrices given by

$$
A_1 = \begin{pmatrix}
2 & 2 & 4 & 0 & 0 & 0 & \ldots & 0\\
2 & 2 & 1 & 1 & 1 & 0 & \ldots & 0\\
0 & & \ldots & \ldots & & & & 0\\
0 & & \ldots & \ldots & & & & 0\\
0 & & \ldots & \ldots & & & & 0
\end{pmatrix}
A_2 = \begin{pmatrix}
1 & 1 & 1 & 1 & 0 & \ldots & & 0\\
1 & 1 & 1 & 1 & 0 & & \ldots & 0\\
2 & 2 & 2 & 2 & 0 & & \ldots & 0\\
0 & & \ldots & \ldots & & & & 0\\
0 & & \ldots & \ldots & & & & 0
\end{pmatrix}
$$

$$
A_3 = \begin{pmatrix}
0 & & & \ldots & \ldots & & & 0\\
0 & & \ldots & \ldots & & & & 0\\
1 & 1 & 1 & 1 & 1 & 0 & \ldots & 0\\
2 & 2 & 2 & 1 & 1 & 0 & \ldots & 0\\
0 & & \ldots & \ldots & & & & 0
\end{pmatrix}
A_4 = \begin{pmatrix}
0 & & & & & & & & & & & \ldots 0\\
0 & & & & & & & & & & & \ldots 0\\
0 & & & & & & & & & & & \ldots 0\\
1 & 1 & 1 & 1 & 1 & 1 & 0 & & & & & \ldots 0\\
2 & 2 & 2 & 2 & 2 & 2 & 1 & 1 & 1 & 1 & 0 & \ldots 0
\end{pmatrix}
\tag{S8}
$$

$$
M = \begin{pmatrix}
 & & & 0_{2\times 14} & & & \\
1 & 1 & 1 & 1 & 0 & \ldots & 0\\
 & & & 0_{11\times 14} & & &
\end{pmatrix}
\tag{S9}
$$

and L_1, L_2, L_3 and L_4 are defined as follows. Let N be the 14×14 matrix with 0s on the diagonal and 1's off the diagonal. Then

$$
\begin{aligned}
L_1 &= \text{diag}\{15,\ 15,\ 17,\ 13,\ 13,\ 13,\ \ldots, 13\} + N \\
L_2 &= \text{diag}\{17,\ 17,\ 17,\ 17,\ 13,\ 13,\ \ldots, 13\} + N \\
L_3 &= \text{diag}\{15,\ 15,\ 15,\ 14,\ 14,\ 13,\ 13,\ \ldots, 13\} + N \\
L_4 &= \text{diag}\{15,\ 15,\ 15,\ 15,\ 15,\ 15,\ 14,\ 14,\ 14,\ 14,\ 13,\ 13,\ 13,\ 13\} + N.
\end{aligned}
\tag{S10}
$$

To see that the added patterns are stable, note that the non-diagonal elements outside of $J^{\max}$ are all less or equal to 2, with the exception of $J^{\max}_{1,7} = 4$. Note that $2\lambda + I > 4$ and $3\lambda + I > 6$. Hence the directions not corresponding to one of the active elements must be stable. For the patterns with two active elements the weakest eigenvalue occurs for the patterns $e_{1,j}$, $j = 6, 7$ and equals $-11 + 2\lambda + I + \mu = -0.8 < 0$. All the other eigenvalues are more negative. For the patterns with three active neurons the situation is similar, the patterns $e_{1,2,j}$, $j = 6, 7$, give the weakest eigenvalue equal to $-(9+3+2)+(3\lambda+I+\mu) = -0.4$. Figure 4 shows a simulation for the required chain (S4).
